# Draft Genome Sequence of *Bacillus respiratorii* VT-16-64, Isolated from the Bronchiolar Alveolar Lavage Fluid of a Patient with Chronic Obstructive Pulmonary Disease

**DOI:** 10.1128/genomeA.00264-17

**Published:** 2017-04-27

**Authors:** Victor Tetz, George Tetz

**Affiliations:** Human Microbiology Institute, New York, New York, USA

## Abstract

We report here the draft genome sequence of *Bacillus respiratorii* VT-16-64, a novel spore-forming bacterium isolated from the bronchiolar alveolar lavage fluid of a patient with chronic obstructive pulmonary disease. The genome was comprised 4,831,386 bp with 4,399 predicted protein-coding genes, including those associated with antibiotic resistance and virulence.

## GENOME ANNOUNCEMENT

*Bacillus respiratorii* VT-16-64 is a spore-forming, motile, Gram-positive, aerobic, and rod-shaped bacterium that, using a combined culture and genetic workflow, was isolated from the bronchiolar alveolar lavage fluid of a patient with chronic obstructive pulmonary disease. *Bacillus* spp. have been implicated in numerous infections (1, 2). The number of *Bacillus* spp. associated with human pathologies has increased dramatically. Along with advances in technology, this allows us to uncover and study the previously unexplored diversity of the *Bacillaceae* family (3–6).

The 16S rRNA gene sequences of *B. respiratorii* VT-16-64 shared 95% similarity with those of various *Bacilli* strains, including *Bacillus oceanisediminis* 2691 and *Bacillus* sp. FJAT-27231.

The whole-genome sequence was obtained using the Illumina HiSeq 2500 sequencing platform (Illumina GAIIx, Illumina, San Diego, CA, USA). Library preparation, sequencing reactions, and runs were carried out according to the manufacturer’s instructions. Reads were assembled *de novo* with SPAdes version 3.5.0 genome assembly software (7). Based on the assembly results, there were 352 scaffolds and the total coverage of the genome was 150-fold. The total genome of *B. respiratorii* VT-16-64 was 4,831,386 bp with a G+C content of 42.7%.

The assembled sequences were annotated using the NCBI Prokaryotic Genome Annotation Pipeline (8). The results indicated that the genome contained 4,399 protein-coding sequences, 69 tRNAs, 14 rRNAs, and four ncRNA operons. Bacterial strains that show <97% 16S rRNA sequence similarity are considered to belong to a new species and can be excluded from DNA–DNA binding (9).

The resistance genes detected in *B. respiratorii* VT-16-64 included transporters of the ABC, MATE, and MFS families, a penicillin-binding protein, and proteins encoding resistance to bacitracin, vancomycin, glyoxalase/bleomycin, and fosfomycin. We also identified the presence of virulence factors, including hemolysin secretion protein D, enterotoxin, proteases, peptidases, phospholipases, and exonucleases, as well as capsular, flagellar, and sporulation proteins (10, 11). In addition, we identified superoxide dismutase, which is considered to possess cancer-inducing properties in some bacteria (12).

Overall, the availability of the present genome sequence will facilitate further analysis of the role of *B. respiratorii* VT-16-64 in chronic obstructive pulmonary disease and other respiratory tract diseases.

### Accession number(s).

The complete genome sequence of *B. respiratorii* VT-16-64 has been deposited in GenBank under the accession number MSRF00000000.
